# Disentangling Many-Body Effects in the Coherent Optical Response of 2D Semiconductors

**DOI:** 10.1021/acs.nanolett.2c01309

**Published:** 2022-06-27

**Authors:** Chiara Trovatello, Florian Katsch, Qiuyang Li, Xiaoyang Zhu, Andreas Knorr, Giulio Cerullo, Stefano Dal Conte

**Affiliations:** † Dipartimento di Fisica, Politecnico di Milano, Piazza L. da Vinci 32, I-20133 Milano, Italy; ‡ Institut für Theoretische Physik, Nichtlineare Optik und Quantenelektronik, Technische Universität Berlin, 10623 Berlin, Germany; § Department of Chemistry, Columbia University, New York, New York 10027, United States

**Keywords:** Transition metal dichancogenides, pump−probe, exciton dynamics, Kramers−Kronig analysis, many-body effects, coherent optical response

## Abstract

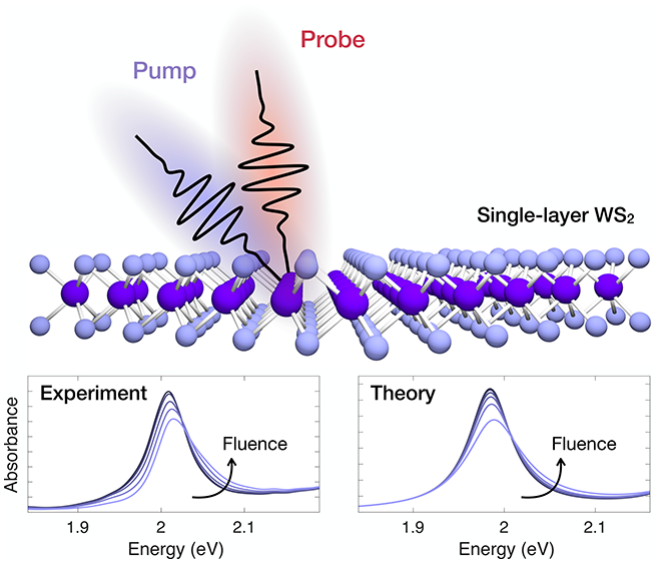

In single-layer (1L) transition metal dichalcogenides, the reduced Coulomb screening results in strongly bound excitons which dominate the linear and the nonlinear optical response. Despite the large number of studies, a clear understanding on how many-body and Coulomb correlation effects affect the excitonic resonances on a femtosecond time scale is still lacking. Here, we use ultrashort laser pulses to measure the transient optical response of 1L-WS_2_. In order to disentangle many-body effects, we perform exciton line-shape analysis, and we study its temporal dynamics as a function of the excitation photon energy and fluence. We find that resonant photoexcitation produces a blue shift of the A exciton, while for above-resonance photoexcitation the transient response at the optical bandgap is largely determined by a reduction of the exciton oscillator strength. Microscopic calculations based on excitonic Heisenberg equations of motion quantitatively reproduce the nonlinear absorption of the material and its dependence on excitation conditions.

Atomically thin transition metal dichalcogenides (TMDs) have received increasing attention because of their optical and electronic properties,^1^ including enhanced light-matter interaction,^2^ strongly bound excitons,^3^ exciton Rydberg states,^4^ multiparticle excitonic complexes,^5−7^ many-body effects,^8^ and chiral optical valley selectivity.^9^ Some of these properties have been also exploited in the realization of prototypical optoelectronic devices with improved performances and decreased size.^10,11^ In view of applications to optoelectronics and photonics, it is of paramount importance to understand the transient optical properties of these materials after pulsed laser excitation.

Transient optical spectroscopy has been extensively used to study exciton scattering processes on the ultrafast time scale.^12,13^ Experimental studies have shown that the formation of excitons in TMDs takes place on a few tens femtoseconds time scale.^14,15^ The exciton population decays with different rate constants, which are the result of several relaxation channels. Although it has been shown that on a tens to hundreds picoseconds time scale the exciton decay dynamics is dominated by interactions with the lattice^16^ (i.e., the exciton–phonon scattering process and the subsequent cooling of phonons due to the energy release to the substrate), the physical origin of exciton dynamics on the subpicosecond time scale is still under debate. In this temporal window, many-body effects leading to energy renormalization and broadening of the excitonic peaks overlap in time with the phase-space filling effect.^17−20^

All of these processes determine the complex line shape of the transient absorption (TA) spectra of TMDs across the bandgap at early time delays (i.e., during and immediately after the temporal overlap of pump and probe pulses) and are difficult to disentangle. In the literature, transient spectral shifts of the exciton resonance have been estimated from TA measurements by different methods.^16,21−27^ Different signs and strengths of the energy shift have been reported depending on pump fluence and photon energy. Several mechanisms have been proposed to describe the renormalization effect upon photoexcitation. Free charge-induced change of the Coulomb screening produces instantaneous band gap and exciton binding energy renormalization, causing opposite shifts of the excitonic peak that partially compensate.^15,18,22^ Optical Stark effect - in the framework of the dressed two-level system picture - has been invoked to explain a change of the sign of the shift.^28^ The extension of this model, including many-body exciton–exciton interactions, has been reported in refs (24) and (29). Finally, coherent biexcitons^17,30^ have been proposed to play a key role in the transient Stark shift, and the experimental signature of their fine structure^31^ has been reported. Despite the large number of works, it is not clear yet how many-body exciton interactions affect the shape and energy shift of the excitonic peaks in the time domain and how these effects change with the energy and the strength of the excitation.

In this work, we measure the broadband transient optical response of 1L-WS_2_ to study the ultrafast dynamics of the A and B excitonic resonances. By using Kramers–Kronig (KK) constrained variational analysis^32^ within the thin-film model approximation,^33^ we retrieve the absorption spectrum as a function of time, and we systematically study its dependence on the pump photon energy and intensity. We find that many-body excitonic effects are strongly enhanced for resonant excitation and result in a transient blue shift of the A excitonic resonance. The shift progressively decreases as the pump is detuned from the resonance and turns into a small red shift when the energy of the pump is on-resonance with the B exciton. We rationalize our results by computing the nonlinear absorption spectra in the coherent limit (i.e., where the pump and probe pulses temporally overlap) using the excitonic Heisenberg equation formalism,^34,35^ which includes the coupling between optically excited excitons and higher-order four-particle correlations, (i.e., biexcitons and exciton–exciton scattering).^36^ The calculated spectra and their dependence on the excitation parameters are in excellent agreement with the experiments.

Our combined experimental–theoretical studies unveil the complex interplay of different energy renormalization, many-body, and exciton population effects in the transient optical response of 1L-WS_2_ by fully disentangling the temporal evolution of bleaching, energy shift, and broadening of the A exciton resonance. These results represent a step forward in the understanding of the coherent optical response of TMDs and go beyond theoretical models for light–matter interaction based on the dressed-atom picture (i.e., in which excitons are treated as noninteracting particles).

The large area 1L-WS_2_ sample on SiO_2_ is prepared using a gold-assisted mechanical exfoliation technique^37^ (see Supporting Information (SI) Supplementary Note 1). The sample is photoexcited with sub-100 fs laser pulses on- and above-resonance with respect to the A exciton (i.e., the optical bandgap), and at variable incident fluence of 1–50 μJ/cm^2^, well below the estimated exciton-Mott transition^21^ (incident fluence >700 μJ/cm^2^, corresponding to an injected carrier density of 1 × 10^14^ cm^–2^). The optical response at the A and B excitons is monitored by a delayed broadband white light probe (see SI Supplementary Note 2).

Figure 1a,b shows the transient reflectivity (*ΔR*/*R*) maps as a function of pump–probe delay and probe photon energy, for excitation resonant to the A and B exciton, respectively. Although the overall appearance of the two maps is similar, upon a close inspection we can capture their qualitative differences around the optical gap within the first few hundred femtoseconds. In both cases, the *ΔR*/*R* signals rise within the temporal resolution of the experiment (i.e., ∼100 fs). For resonant excitation, the *ΔR*/*R* spectrum at 0 fs (Figure 1c), that is, at the temporal overlap of pump and probe pulses, is characterized by a derivative-like shape, whereas for above-resonance excitation (i.e., resonant to B) the signal at zero delay (Figure 1d) presents a double sign change centered at the gap. In the latter case, the shape of the transient spectra does not significantly change at longer delays (see the *ΔR*/*R* spectrum at 500 fs). For excitation at the bandgap the lower energy part of the spectrum quickly changes after about 200 fs, as it can be seen from the sign flip (from negative to positive) of the *ΔR*/*R* spectrum at 500 fs below 1.95 eV.

**Figure 1:**
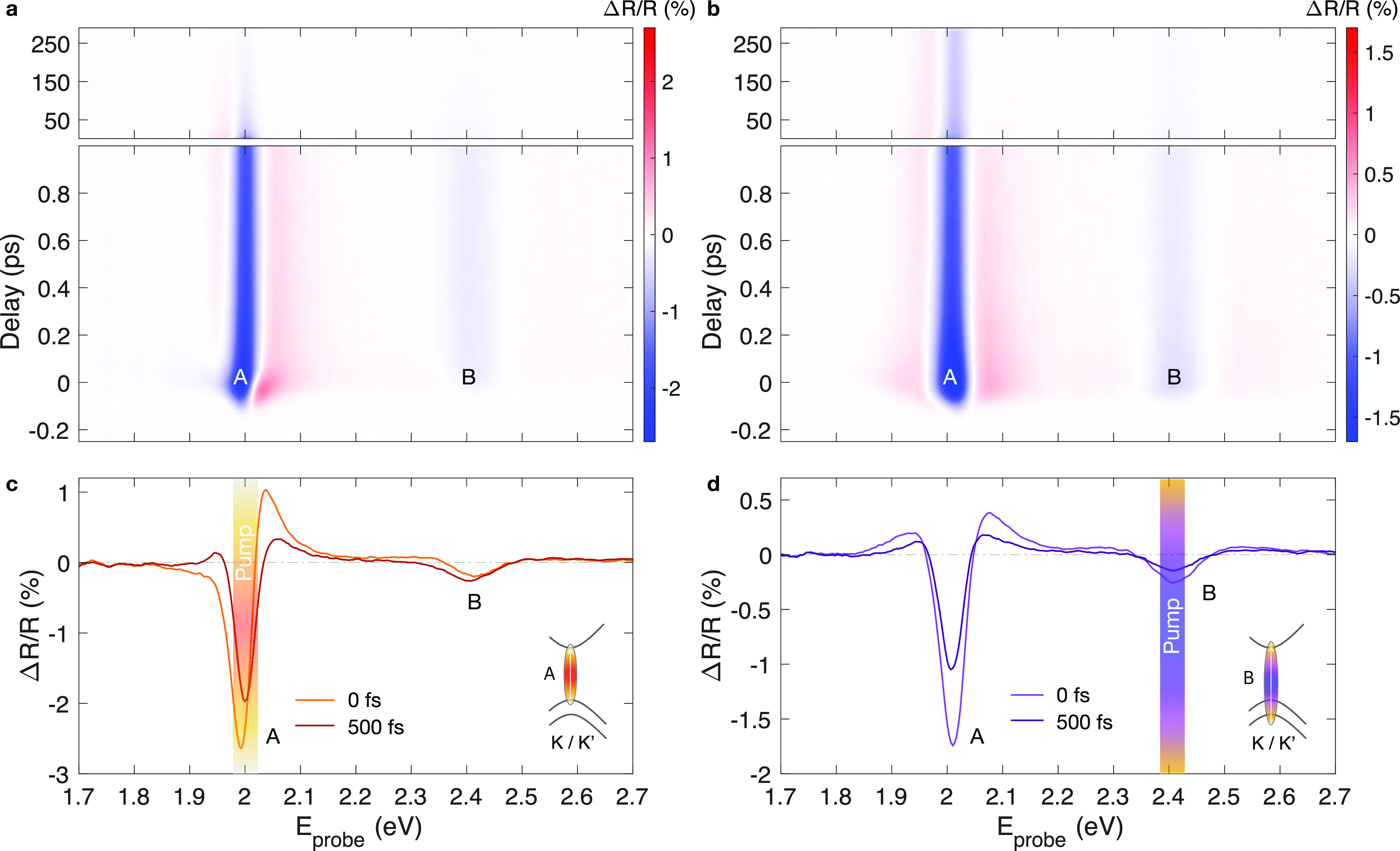
Pump photon energy dependent transient optical response of 1L-WS_**2**_. (a,b) Transient reflectivity maps of 1L-WS_2_, as a function of delay and probe photon energy (*E*_probe_), following (a) resonant excitation at the optical bandgap (2.00 eV, incident fluence 10 μJ/cm^2^) and (b) excitation at the B exciton photon energy (2.43 eV, incident fluence 35 μJ/cm^2^). (c,d) Extracted *ΔR*/*R* spectra at 0 and 500 fs delay for (c) on- and (d) above-resonance excitation, respectively.

In order to translate the *ΔR*/*R* spectrum into an evolution of the excitonic peak, we retrieve the absorption spectrum with and without pump excitation by exploiting the KK constrained variational method^32^ within the thin film approximation analysis,^33^ similar to the approach adopted in refs.^2,23^

The static absorption spectrum and the nonequilibrium absorption spectra at the time delay for which the excitonic bleaching is maximum are reported in Figure 2a,b for different excitation photon energies. For resonant excitation (Figure 2a), we observe a dramatic change of the A exciton peak, which is the result of several concomitant effects: a quenching of the exciton oscillator strength, a blue shift by ∼6 meV and an asymmetric line broadening. All of these transient effects are less evident for the B exciton peak since it is characterized by large broadening and small oscillator strength (see Figure S9 for detailed information on the B exciton dynamics). All of the spectra are well reproduced by a fitting function consisting of the sum of two Lorentz oscillators (for the A and B exciton) and a polynomial background (see SI Supplementary Note 4). The temporal evolution of the Lorentz oscillator parameters of the A exciton peak is reported in Figure 2c–e. Similar dynamics of the exciton line shape has been previously reported for resonant excitation.^24^ In that study, the transient energy shift has been described as a coherent process and rationalized in terms of optical Stark effect due to dressing of the excitonic transition by the ultrashort pump pulse. Our measurements are not consistent with that interpretation, because they show that the energy shift persists over a time scale longer than the temporal overlap of the pump and the probe pulses. The decay time scales can be extracted using a fitting function (as explained in SI Supplementary Note 7). The *E*_XA_ dynamics is chacterized by a first decay of 55 ± 4 fs and a second one of 1.4 ± 0.6 ps, both resolved by our experiment. This fast decay dynamics is compatible with the relaxation of the excitonic population due to several decay channels on the subpicosecond time scale.^38^

**Figure 2:**
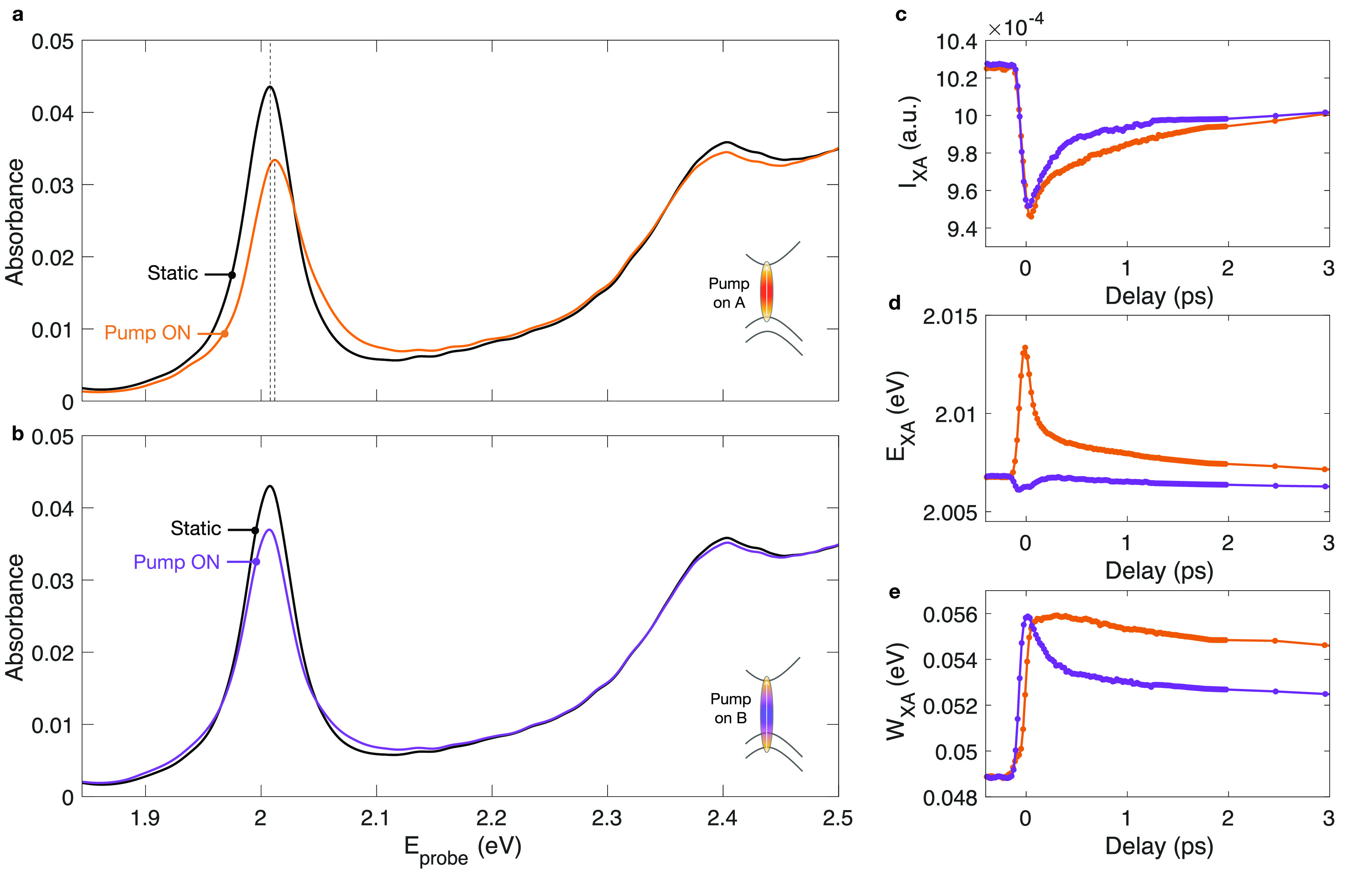
Time-dependent exciton line shape analysis. (a,b) Measured optical absorbance spectra of 1L-WS_2_ with and without photoexcitation for (a) resonant and (b) above-resonance photoexcitation. We set the pump fluences such that the maximum of the transient signal for both excitation photon energies is nearly the same. (c–e) Temporal evolution of the A excitonic resonance parameters: (c) intensity, (d) peak energy, and (e) line width for on- (orange) and above-resonance (violet) pump photon energy.

The other two fitting parameters (*I*_XA_ and *W*_XA_) display an instantaneous variation and they all decay on the hundreds ps time scale (see SI Supplementary Note 6). In this temporal window, the transient optical properties of the material are expected to be dominated by interactions with phonons.^16^

The retrieved out-of-equilibrium absorption spectrum for nonresonant excitation, reported in Figure 2b, shows a different shape. Interestingly, the energy renormalization of the A excitonic peak is reduced by an order of magnitude and its sign reversed (i.e., energy redshift) with respect to the resonant excitation. At this excitation photon energy, the broadening and the bleaching of the peak are the dominating effects. The sign of the transient energy shift is in agreement with previous measurements performed on WS_2_.^21^ We note that in this previous study a giant energy renormalization of the excitonic peak was observed for fluences of 800 μJ/cm^2^, above the exciton-Mott transition (i.e., over an order of magnitude higher than the maximum fluence used in our measurements). The dynamics of the fitting parameters, obtained by repeating the previous analysis, are reported in Figure 2c–e.

Figure 3a,b shows the measured fluence-dependent absorption spectra at a fixed time delay, corresponding to the maximum of the TA signal. In addition to the stronger energy shift of the A exciton peak for on-resonant excitation (see SI Supplementary Figure 4), we also find that the A exciton exhibits an instantaneous broadening characterized by a pronounced deviation from the symmetric Lorentzian line shape at increasing fluences (see the normalized and shifted spectra in the inset of Figure 3a). The strong asymmetry of the excitonic peak is observed almost instantaneously and the original Lorentzian shape is quickly recovered within few picoseconds (see SI Supplementary Note 5). Conversely, when the material is excited above the excitonic resonance (Figure 3b), no asymmetry is observed in the broadening dynamics of the peak. The physical process behind the transient line width increase is called excitation-induced dephasing, and it has been previously observed in several semiconductor nanostructures measured by coherent optical spectroscopy experiments.^39,40^ This effect has been microscopically described in term of different excitonic scattering processes such as exciton-continuum and two-pair-continuum scattering.^41^ In principle, exciton–exciton and exciton–phonon scattering processes can contribute to transient broadening of the exciton peak, leading to an asymmetric change of the excitonic resonance. The coupling between exciton and the phonon continuum determines the formation of high-energy sidebands, which results in an asymmetric exciton line shape as observed in the static absorption spectra of TMDs at room temperature.^42^ In this case, these features are attributed to the absorption and emission of optical and acoustic phonon modes. The formation of phonon side bands has been directly measured in time-resolved photoluminescence experiments^43^ but the characteristic time scale of this process is on the order of some picoseconds at low temperature, that is, much slower than the asymmetric line width broadening dynamics observed in our measurements. This result supports the dominant role of exciton–exciton scattering in the exciton induced dephasing process. However, at room temperature, exciton–phonon coupling is much faster and might also contribute to the broadening dynamics.

**Figure 3:**
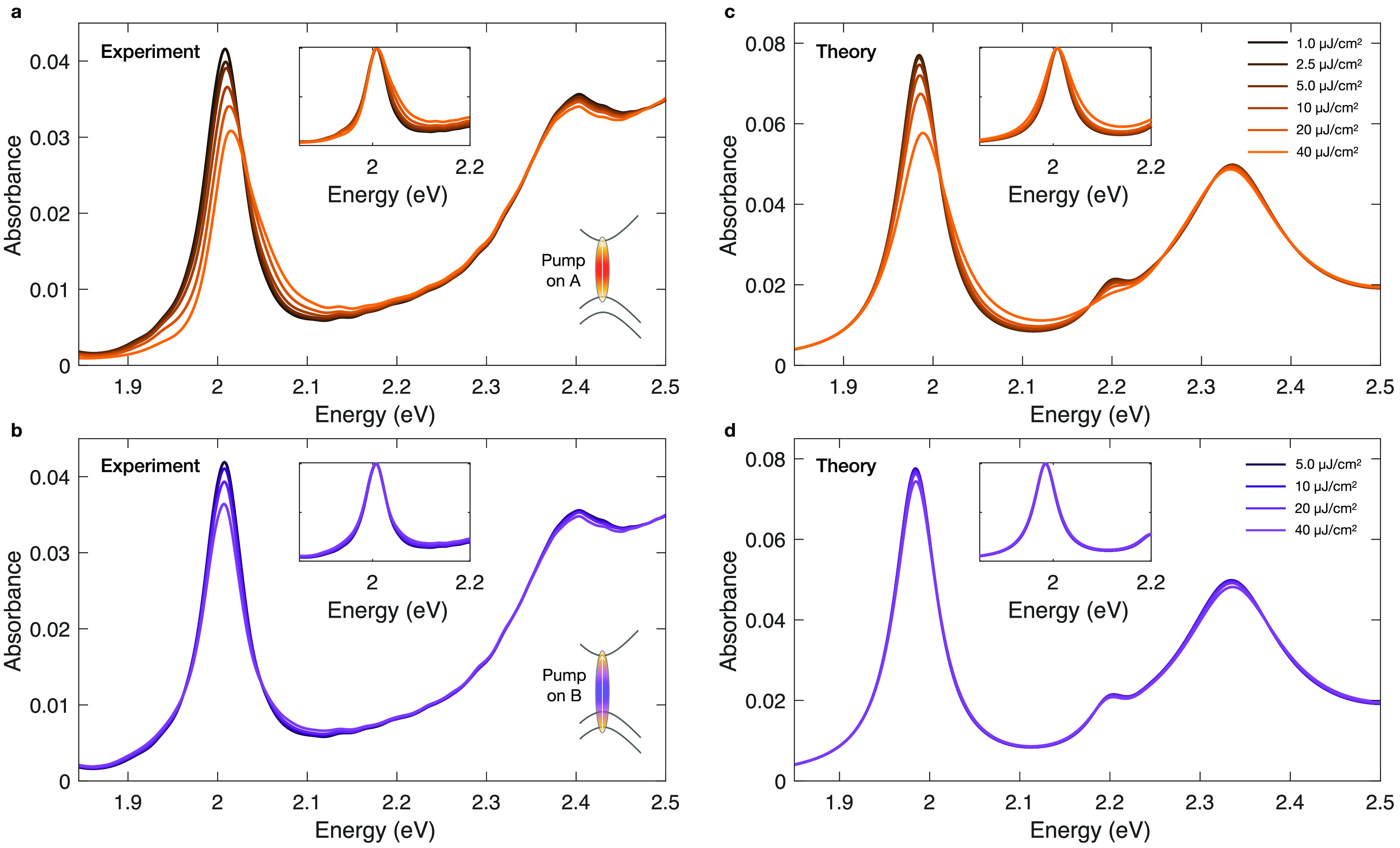
Optical-pump-fluence dependent absorption of 1L-WS_**2**_. (a,b) Measured and (c,d) calculated pump fluence-dependent absorbance spectra of 1L-WS_2_ for (a,c) resonant and (b,d) nonresonant excitation. (Insets) Normalized and shifted fluence-dependent spectra.

In order to provide a physical explanation of all the observed transient modifications of the excitonic transition, we performed calculations based on excitonic Heisenberg equations of motion. This microscopic approach includes phase-space filling and many-body effects, which are expected to take place upon photoexcitation. In particular, the model considers two exciton–exciton interaction effects: Coulomb coupling between optically addressable excitons in the same valley and intervalley coupling between excitons in different valleys. Four-particle correlations such as biexcitons and the associated exciton–exciton scattering continua are also included in the calculation.

Our approach consists in first solving the Wannier equation,^44^ which gives access to the exciton binding energies ϵ_x,λ_ along with the exciton wave functions φ_λ,***q***_^44^, and allows one to calculate the excitonic polarization *P*_λ_. λ denotes a compound index incorporating the exciton state 1s, 2s, ..., the high-symmetry point K^(′)^, and the spins ↑, ↓ of the electrons and holes forming the exciton.^45^ Then the solution of the Schrödinger equation for two electrons and two holes provides the access to intravalley exciton–exciton scattering continua with energy above the exciton energy and bound intervalley biexcitons. Nonlinear optical properties of the TMD are accessed by calculating the dynamics of the excitonic interband polarization *P*_λ_1__ in the coherent limit within the formalism of the Heisenberg equations of motion
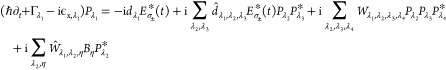
1The left-hand side of eq 1 describes optical oscillations with the energy ϵ_x,λ_1__and a phonon-mediated damping rate Γ_λ_1__.^46,47^ The optical selection rules^48^ are comprised in the excitonic transition matrix element *d*_λ_1__. The radiative dephasing is determined by self-consistently treating the light–matter interaction,^49,50^ that is, by solving the Maxwells’s and TMD Bloch equations at the same time and by taking into account the incident field and its renormalization at the position of the sample due to the generated interband polarization. The second term on the right-hand side of eq 1 represents Pauli blocking, i.e., a phase space filling effect.^51^ The third line of eq 1 describes nonlinear exciton–exciton interactions on a Hartree–Fock level, and the last line characterizes the coupling between excitons and biexcitonic two-electron and two-hole Coulomb correlations *B*_η_. Here, the index η serves as a compound index and includes the high-symmetry points and spins of the two electrons and two holes. The solution of the biexcitonic Schrödinger equation for two electrons and two holes accesses bound biexcitons as well as exciton–exciton scattering continua which set in at twice the exciton energy.^52−55^ The associated Heisenberg equations of motion for bound biexcitons as well as continuous exciton–exciton scattering states *B*_η_ characterize damped (Γ_*xx*_) oscillations (energy ϵ_xx,η_) which are driven by two excitons *P*__λ__*P*_λ_2__ mediated by Coulomb interactions (*Ŵ*_η,λ_1_,λ_2__)
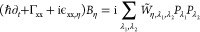
2

Although the theory is developed for the whole ensemble of excitonic states, we restrict the calculation to nearly the same spectral region covered by the experiments and we include the lower energy Rydberg states (i.e., 1s and 2s) of the A and B excitons. Nonlinear absorption spectra are calculated at zero delay time, by solving the TMD Bloch equations, the equations of motion for the exciton and biexciton amplitudes and the Maxwell’s equations.^44,49^ Dynamical Coulomb screening effects due to optical photoexcitation of excitons are included in our simulations; these effects renormalize the exciton binding energy and the exciton–exciton interaction.

The calculated absorption spectra at increasing excitation fluences are reported in Figure 3c,d for pumping resonant to the A and B exciton, respectively, close to the experimental spectra measured with same excitation condition for better comparison. All of the spectra are computed for linearly polarized excitation, resulting in a simultaneous photoinjection of excitons in K and K′ valleys. The calculated spectra reproduce quantitatively the experimental curves and their fluence and excitation energy dependence. For resonant excitation, the A exciton oscillator strength is progressively reduced for increasing fluences because of the Pauli blocking effect and the spectral weight redistribution due to Coulomb mediated exciton–exciton scattering. These two processes are described, respectively, by the second term, the third and fourth terms on the right-hand side of eq 1.

The Coulomb coupling between excitons leads also to the formation of a biexciton peak which is energetically (∼11 meV) below the A exciton. The biexciton binding energy has been estimated by solving the four-particle Schrodinger equation and is a factor two smaller than the recently measured experimental value.^56^ This feature is not clearly visible in the experimental spectra because it is obscured by the large line width of the A exciton peak. Signatures of biexcitonic features, characterized by a fine structure, have been observed in the coherent transient optical response of high-quality TMD samples with line widths approaching the homogeneous limit.^7,31^

The sign and the fluence dependence of the A exciton peak shift are also well reproduced by theory. The blue shift of the peak is the result of complex interplay between different effects: the Coulomb interaction between excitons within a Hartree–Fock approximation results in a blue shift while the coupling between excitons and two-electron and two-hole Coulomb correlations induces a smaller shift of opposite sign. Another important feature that is captured by the theoretical formalism is the line width broadening characterized by an asymmetry degree which increases with the incident fluence. Exciton–exciton and exciton–biexciton interaction processes contribute not only to the energy renormalization of the peak but also redistribute the excitonic spectral weight to higher energies. We stress that in order to model the line width broadening and its power dependence seen in the experiments, we did not need to consider additional disorder effects such as the inhomogeneous broadening of the excitonic resonances.

Qualitatively, the same discussion holds also true for the A exciton when pumping resonant to the B exciton, as shown in Figure 3d. However, the reduction of oscillator strength, blue shift, and broadening are much weaker compared to resonant excitation of the A exciton depicted in Figure 3b. The shift of the A excitonic resonance occurs mainly due to the Hartree–Fock interactions of the excitons, appearing as products of the excitonic transitions *P*_λ_ in eq 1, line 3. The efficiency of the optical excitation of these transitions depends on the detuning from their resonance and their dipole coupling element. For a detuned excitation of higher lying states (i.e., 2s, continuum), the pump efficiency for the 1s A-exciton is decreased. Similarly, when the excitation is tuned on-resonance with the 1s states, the off-resonantly involved dipole coupling matrix elements of higher lying states are weaker. Both effects decrease the number of excited excitons and therefore the exciton–exciton interaction at similar pump fluence. Therefore, for increased excitation detuning, the efficiency of the involved Hartree–Fock contributions decreases at the expense of the (also decreasing) Pauli blocking. In addition, all Hartree–Fock matrix elements involve convolutions of the wave functions of the excited excitons. The reduced strength of Hartree–Fock contributions at increased detuning is explained as a result of destructive interference due to an increasing number of knots in the wave functions occurring in the interaction integrals.

In conclusion, we have measured the transient optical response of 1L-WS_2_ at the optical bandgap following on- and above-resonance photoexcitation, at intensities well below the exciton-Mott transition. By using Kramers–Kronig constrained variational analysis and exciton line-shape analysis, we have fully disentangled and quantified the contributions of absorption bleaching, energy shift, and broadening to the transient line width of the A exciton. We find that resonant photoexcitation produces a transient blue shift of the resonance, which originates from the dominant Coulomb Hartree–Fock contributions, that is, the interplay of bandgap renormalization and mean-fields effectively experienced by the optically excited excitons. This is also indicated by the peculiar asymmetric shape of the exciton line width at high photon energies in the first picoseconds after photoexcitation. On the contrary, for above-resonance photoexcitation the transient optical response is almost entirely dominated by a transient reduction of the exciton oscillator strength due to a phase-space filling effect, while energy renormalization is one order of magnitude smaller and with opposite sign (red shift). Our experimental findings are corroborated by microscopic calculations based on excitonic Heisenberg equations of motion, that quantitatively reproduce the out-of-equilibrium absorption of the monolayer and its dependence on the excitation fluence and photon energy.

Our results provide a more refined understanding of the transient optical response of 2D semiconductors and give important insights into the complex interplay between many-body correlations and excitonic interactions which govern the nonequilibrium response of these materials. As a natural prosecution of this work, similar experiments could be performed on high-quality hBN encapsulated TMD samples in order to elucidate how Coulomb many-body interactions and also electron–hole exchange interaction determine the nonlinear optical response of different excitonic complexes which are energetically close to the neutral exciton (i.e., trions and biexcitons).^57^

## Supporting Information Available

Sample preparation; experimental setup; Kramers–Kronig constraint variational analysis; fitting the nonequilibrium absorbance spectra; exciton asymmetric line shape Pump–probe data; Fitting the temporal dynamics; Theoretical calculations (PDF)
