# Supplementary material for: Disentangling Many-Body Effects in the Coherent Optical Response of 2D Semiconductors
Source: Nano Lett. 2022 Jun 27;22(13):5322–9. doi: 10.1021/acs.nanolett.2c01309 (PMC9284612; doi:10.1021/acs.nanolett.2c01309)
Supplement: Supplementary file 1 — nl2c01309_si_001.pdf [file nl2c01309_si_001.pdf]

Supplementary Information: Disentangling many-body effects in the coherent optical response of 2D semiconductors

Chiara Trovatello,† Florian Katsch,‡ Qiuyang Li,¶ Xiaoyang Zhu,¶ Andreas Knorr,‡ Giulio Cerullo,† and Stefano Dal Conte*,†

†*Dipartimento di Fisica, Politecnico di Milano, Piazza L. da Vinci 32, I-20133 Milano, Italy*

‡*Institut für Theoretische Physik, Nichtlineare Optik und Quantenelektronik, Technische Universität Berlin, 10623 Berlin, Germany*

¶*Department of Chemistry, Columbia University, New York, New York 10027, USA*

E-mail: stefano.dalconte@polimi.it

Supplementary Note 1: Sample preparation

Large area monolayer WS_2 is mechanically exfoliated from a single crystal bulk WS_2 (HQ graphene). The large area exfoliation is based on a gold-assisted technique in which a layer of gold is deposited on the bulk WS_2 crystal and it is subsequently exfoliated using a thermal release tape. The tape carries a large area single crystal monolayer on the contact surface, which is then transferred onto a transparent 200 µm-thick SiO_2 substrate. The thermal release tape is removed by heating up at 130°C, and the residues are removed by using acetone and O_2 plasma treatment. Finally, a gold etchant solution (KI 99.9% and I_2 99.99% in DI water) is used to dissolve the gold layer. This exfoliation technique produces monolayers of millimetric size, whose lateral dimension is only limited by the size of the original bulk crystal. The optical quality of the monolayers is comparable to those obtained from standard scotch tape exfoliation.[1]

Supplementary Note 2: Experimental setup

The source of the pump-probe measurements is an amplified Ti:Sapphire laser (Coherent Libra II) emitting 100 fs pulses at 1.55 eV, with an average power of 4W, and a repetition rate of 2 kHz. The probe beam is obtained by white light generation, focusing the fundamental beam at 1.55 eV onto a 2-mm-thick sapphire plate. The probe fluctuations are as low as 0.2%. The pump beams at 2.00 eV ($\pm 0.016\,$eV) and 2.48 eV ($\pm 0.024\,$eV), respectively, are generated seeding a visible non-collinear optical parametric amplifier. The pump is mechanically modulated by a chopper at 1 kHz. When focused on the sample, pump and probe beams have gaussian spot sizes with FWHM of $\sim 250\,\mu$m and $\sim 180\,\mu$m, respectively. Pump and probe are linearly polarized and have perpendicular polarizations. Both the beams are focused non-collinearly on the sample with a relative incidence angle less than 5 degrees. The reflected probe light is dispersed by a grating on a silicon photodiode array, working at the full laser repetition rate and ensuring a detection sensitivity of $10^{-4} - 10^{-5}$. All the pump-probe maps are corrected for the chirp introduced by white light generation in the post-processing analysis by selecting from the map several temporal traces at different probe wavelengths. For each trace the temporal zero has been carefully estimated from the temporal onset of the almost instantaneous build-up dynamics at different probe wavelengths. Temporal zeros vs probe energies curves have been modelled by a low-order polynomial function.

Supplementary Note 3: Kramers-Kronig constraint variational analysis

Transient absorption spectroscopy measures small photo-induced absorption changes in the material under investigation. Such changes are quantified by recording the probe spectrum with and without photo-excitation by the pump pulse. Depending on the geometry of the experiment one can detect the probe light transmitted/reflected by the sample, and compute the differential transmissivity $\Delta T/T$ or reflectivity $\Delta R/R$. In this work we measure $\Delta R/R$ from 1L-WS_2 on SiO_2:

$$\Delta R/R(\omega,\tau) = \frac{R_{\mathrm{ON}}(\omega,\tau) - R_{\mathrm{OFF}}(\omega)}{R_{\mathrm{OFF}}(\omega)} \tag{1}$$

where R_{ON} and R_{OFF} are the reflected probe spectra with (ON) and without (OFF) the pump excitation, respectively, and τ is the delay between pump and probe pulses. In order to disentangle the photo-induced bleaching, shift and broadening of the excitonic peaks we need to isolate $R_{\mathrm{ON}}(\omega,\tau)$, i.e., the reflectivity of the bare monolayer modified by the pump excitation:

$$R_{\mathrm{ON}}(\omega,\tau) = R_{\mathrm{OFF}}(\omega)(\Delta R/R(\omega,\tau) + 1) \tag{2}$$

While $\Delta R/R(\omega,\tau)$ is directly measured in the pump-probe experiment, $R_{\mathrm{OFF}}(\omega)$ - the reflectivity of the bare monolayer - can be retrieved from reflectivity contrast measurements. Our analysis indeed starts from the measurement of the reflectivity contrast of 1L-WS_2 on a 200 µm-thick SiO_2 substrate:

$$R_{\mathrm{contrast}}(\omega) = \frac{R_{\mathrm{sample}}(\omega) - R_{\mathrm{substrate}}(\omega)}{R_{\mathrm{substrate}}(\omega)} \tag{3}$$

If the thin film is placed on a semi-infinite dielectric substrate with real refractive index n_s,

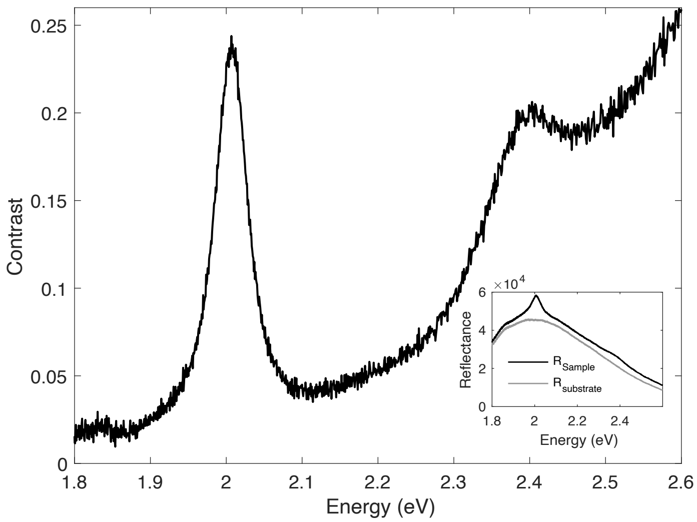

Figure S1: Constrast. Equilibrium reflectivity contrast of 1L-WS_2 on SiO_2.

i.e., the case of SiO_2, the absorbance α is related to the contrast R_{contrast}[2] as:

$$\alpha(\omega) = R_{\text{contrast}}(\omega)\frac{n_s^2 - 1}{(n_s + 1)^2} \tag{4}$$

The thin film approximation provides an analytical expression for absorbance α, reflectance R and transmittance T of the bare monolayer as a function of the dielectric function ϵ,[3] which can be expressed as:

$$\alpha(\omega) = \frac{\frac{4\omega d}{c}\epsilon_2}{(1 + n_s + \frac{\omega d}{c}\epsilon_2)^2 + (\frac{\omega d}{c}(\epsilon_1 - 1))^2} \tag{5}$$

$$R(\omega) = \frac{(1 - n_s - \frac{\omega d}{c}\epsilon_2)^2 + (\frac{\omega d}{c}(\epsilon_1 - 1))^2}{(1 + n_s + \frac{\omega d}{c}\epsilon_2)^2 + (\frac{\omega d}{c}(\epsilon_1 - 1))^2} \tag{6}$$

$$T(\omega) = \frac{4n_s}{(1 + n_s + \frac{\omega d}{c}\epsilon_2)^2 + (\frac{\omega d}{c}(\epsilon_1 - 1))^2} \tag{7}$$

where n_s is the refractive index of the substrate (for fused silica $n_s = 1.46$), d is the monolayer thickness (for 1L-WS_2 $d = 0.67\,\text{nm}$), ϵ_1 and ϵ_2 are real and imaginary part of the dielectric function ϵ ($\epsilon = \epsilon_1 + i\epsilon_2$). In order to extract ϵ from the monolayer absorbance α within

the thin-film approximation, we implement a Kramers-Kronig (KK) constrained variational analysis.[4] This method merges capability of the KK approach to extract information about the spectrum of the dielectric function based on a functional model, with the robustness of the least-square fitting. Briefly, it corresponds to the limiting case of the standard multi-oscillator modeling, with a number of Drude-Lorentz oscillators that is so large to approach the number of experimental points in the spectrum. In this way, while preserving the KK constraints between ϵ_1 and ϵ_2, the analysis essentially becomes model-independent. We construct ϵ as the sum of N=70 Drude-Lorentz oscillators, with equispaced peak energy E_k in the range $1.7\,\text{eV} \leq E \leq 2.6\,\text{eV}$ and fixed broadening $\gamma_k = 35\,\text{meV}$:

$$\epsilon(\omega) = \epsilon_\infty + \sum_{k=1}^{N} \frac{E_{0,k}^2}{E_k^2 - E^2 - iE\gamma_k} \tag{8}$$

where E is the energy, $E_{0,k}$ is the plasma energy of the oscillator k and ϵ_∞ is a constant term accounting for the high energy electronic transitions. Upon fitting $\alpha(\omega)$ to ϵ (Eq. 5), we retrieve ϵ_1 and ϵ_2 (Fig. **S2**).

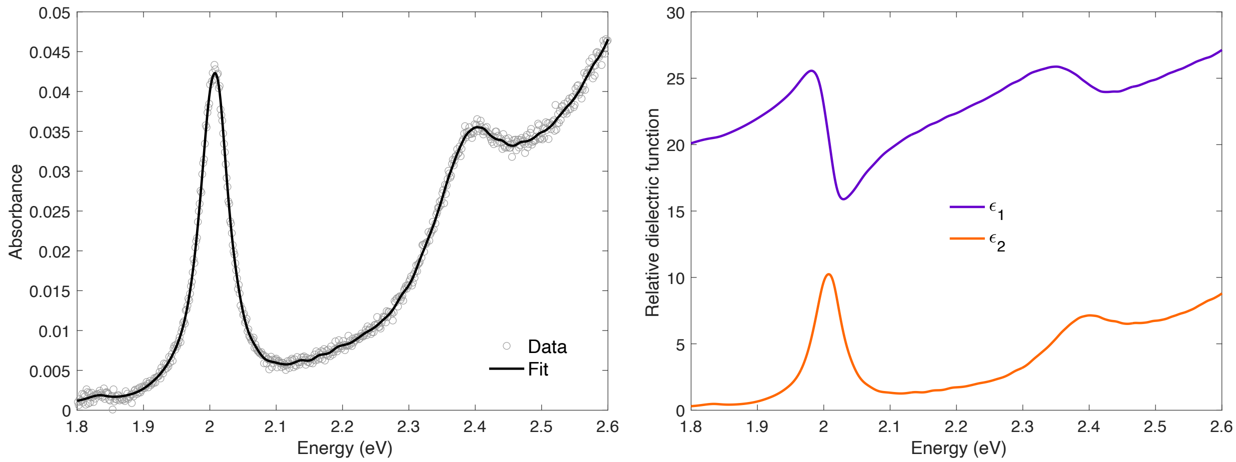

Figure S2: Equilibrium relative dielectric function. Static absorbance of 1L-WS_2 retrieved from the measured contrast and fitting curve to Eq. (8). Extracted quilibrium relative dielectric function ϵ using KK constraint analysis.

With the equilibrium dielectric function we can now compute $R_{\text{OFF}}(\omega)$ and extract $R_{\text{ON}}(\omega, \tau)$ from the pump-probe signal $\frac{\Delta R}{R}(\omega, \tau)$. By reiterating the above mentioned fitting procedure,

we access the *non-equilibrium* dielectric function and we retrieve the *non-equilibrium* absorbance $\alpha_{\mathrm{ON}}(\omega, \tau)$.

Supplementary Note 4: Fitting the non-equilibrium absorbance spectra

In order to track the photo-induced changes in the non-equilibrium absorption spectra of 1L-WS_2 we fit the extracted absorbance $\alpha_{\mathrm{ON}}(\omega, \tau)$ to a sum of two Lorentzian peaks (A and B excitonic resonances) and a polynomial background. Each Lorentzian peak L_k as a function of the energy E can be written as:

$$L_k(E) = E_{0,k} \frac{\frac{\gamma_k}{2}}{(E - E_k)^2 + (\frac{\gamma_k}{2})^2} \tag{9}$$

where $E_{0,k}$ is the oscillator amplitude, γ_k is the linewidth and E_k is the peak energy. During the fit only the parameters of the two excitons can vary, i.e., intensity, broadening and energy, while the polynomial background - extracted from the static absorbance - is kept fixed.

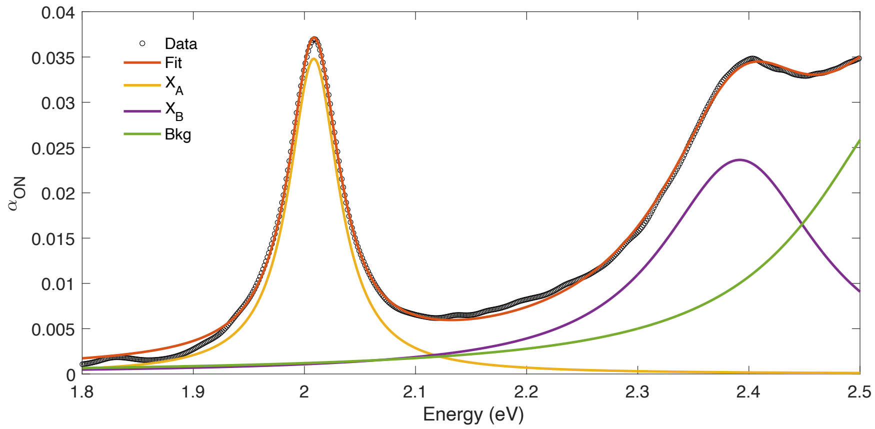

Figure S3: Fitting procedure. A representative non-equilibrium absorbance spectrum $\alpha_{\mathrm{ON}}(\omega, \tau)$ along with the fitting curve.

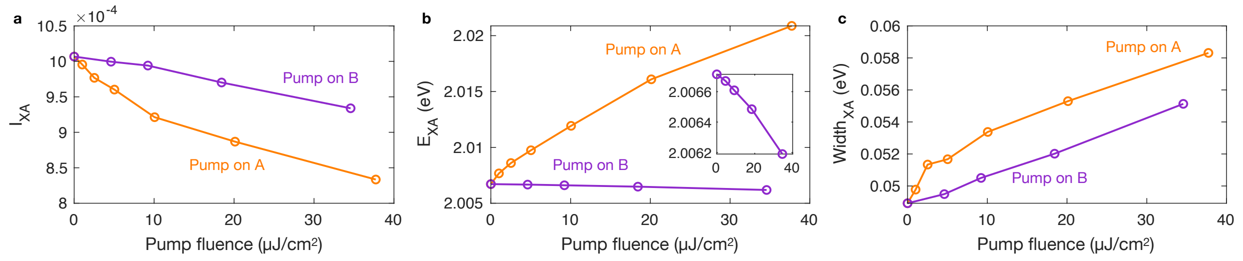

Figure S4: Optical-pump-fluence dependent absorption of 1L-WS_2. Fluence-dependent A exciton parameters for resonant (orange) and non-resonant (violet) photo-excitation: (a) intensity, (b) peak energy and (c) linewidth, for both on- (orange) and above-resonance (violet) pump photon energy obtained from the fit the experimental absorbance. The values at zero pump fluence are extracted from the static absorption spectrum.

Supplementary Note 5: Exciton asymmetric lineshape

When 1L-WS_2 is photoexcited at photon energy resonant with the optical bandgap (i.e., 2 eV), the A exciton resonance displays a strongly asymmetric lineshape. In order to quantify the asymmetry of the peak, the photo-induced change in the non-equilibrium absorption spectra $\alpha_{ON}(\omega, \tau)$ around the A exciton is fitted with a Lorentz oscillator (Eq. 9), but with an energy-dependend linewidth[5] $\gamma(E)$:

$$\gamma(E) = \frac{2\gamma_0}{1 + e^{a(E-E_0)}} \tag{10}$$

where γ_0 is a constant broadening and $a[\mathrm{eV}^{-1}]$ is the asymmetry parameter. If $a = 0$, $\gamma(E) = \gamma_0$, i.e., the case of symmetric broadening. In order to have an intuitive understanding of the asymmetry-dependent lineshape for negative and positive values of a, in Fig. **S5** we plot the Lorentz oscillators with $a > 0$ and $a < 0$, on top of the symmetric reference ($a = 0$).

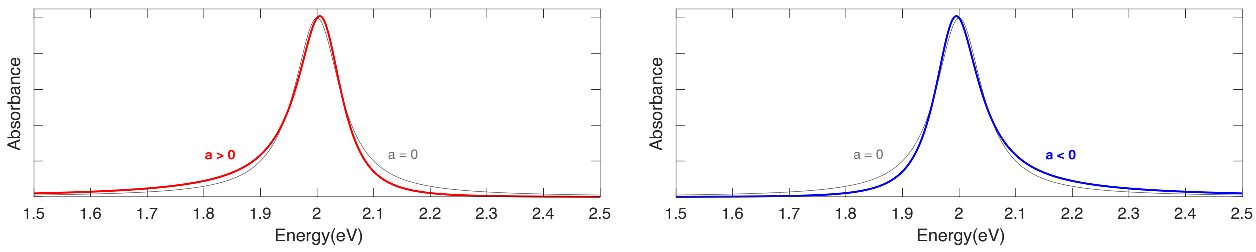

Figure S5: Asymmetric lineshape. Representative asymmetric Lorentzian peaks. For $a > 0$ the peak extends towards the red, while for $a < 0$ the tail of the peak is towards the blue.

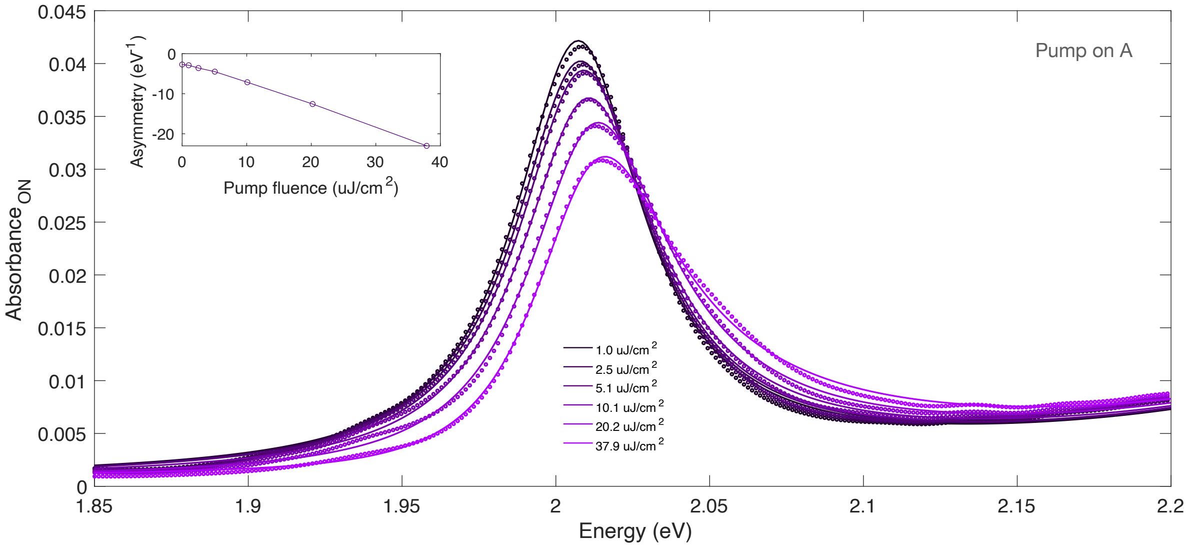

Figure S6: Fitting of the A exciton asymmetric lineshape. Pump fluence-dependent absorbance spectra for resonant excitation. The tail of the A exciton peak extends towards high energies. The asymmetric lineshape fitting curves (lines) display a negative asymmetric parameter, $a < 0$ (see the top left insets for the fluence-dependence of the extracted asymmetric parameter (a)).

Figure **S6** shows the fluence dependent absorbance spectra, which are associated with a pronounced asymmetric linewidth extending on the high-energy side of the A exciton peak. This nonlinear effect originates from many-body interactions that redistribute the oscillator strength, i.e. Coulomb coupling among excitons, biexcitons below the exciton energy, and exciton-exciton scattering continua above the exciton energy.[6] The experimental data (dots) are fitted using an asymmetric Lorentzian peak (line). As the fluence increases the tail extends more to the blue ($a < 0$), see the extracted asymmetric parameter a in the top left inset.

Figure **S7**a-b show the time dependent absorbance spectra of 1L-WS_2 photoexcited (a) on- and (b) above-resonance in the early temporal delays, i.e., within 100 fs from the temporal overlap of pump and probe pulses. In Fig. **S7**c we report the time-dependent asymmetry parameter a at increasing resonant pump fluences. The short decay time of the transient asymmetry of the A exciton peak originates fromKatsch2020 exciton-exciton interaction.

Conversely, when the material is non-resonantly excited (Fig. **S7**b), a much weaker asymmetry is observed in the broadening dynamics of the peak. For comparison, in Fig.

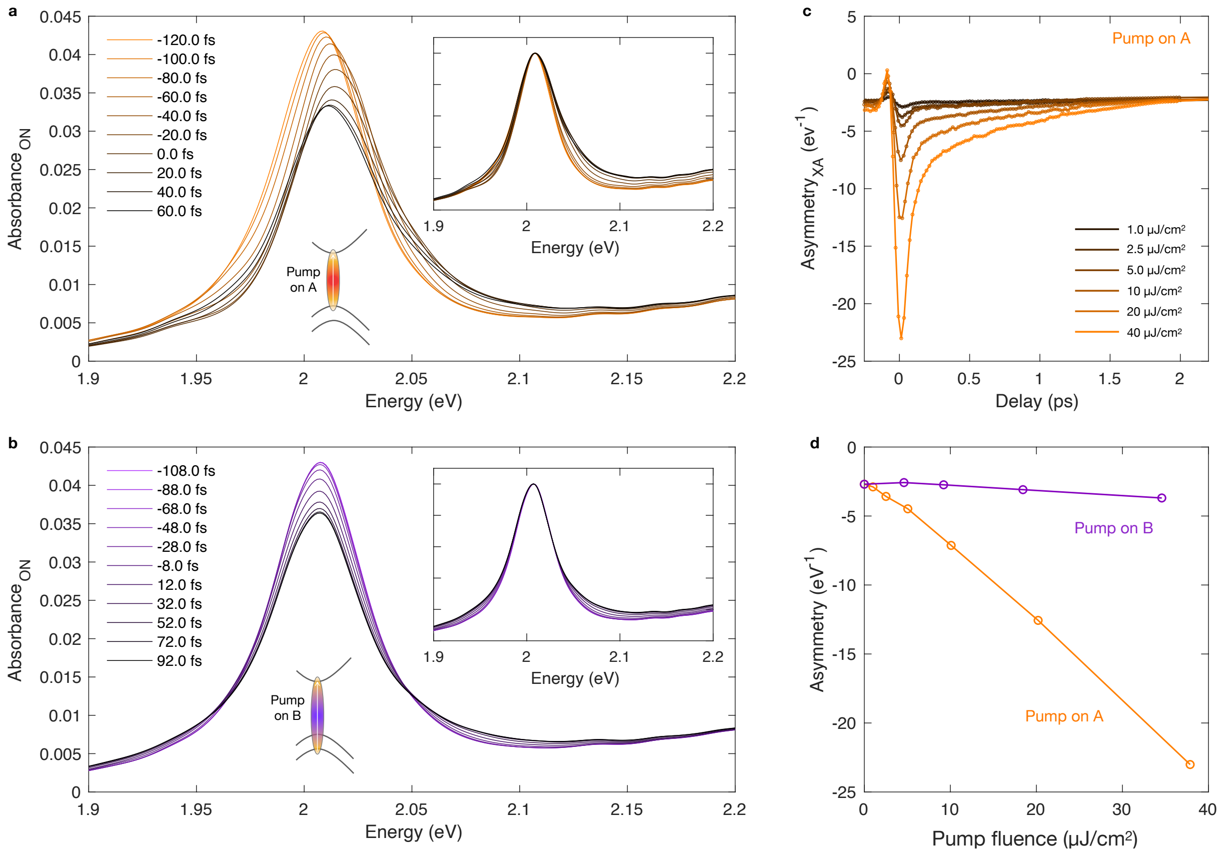

Figure S7: Time- and fluence-dependent asymmetric lineshape of the A exciton.. (a-b) Time dependent absorbance spectra for (a) resonant excitation at 2.00 eV and (b) non-resonant excitation, i.e., 2.43 eV. (c) Time-dependent asymmetric parameter extracted from the fit of the lineshape analysis of the A exciton with an asymmetric lorentzian peak. (d) Fluence-dependent asymmetric parameter of the A exciton for resonant (orange) and non-resonant (violet) photo-excitation. The value at null pump fluence is extracted from the static absorption spectrum.

S7d we report the fluence-dependent strength of the asymmetry parameter. The asymmetry of the A exciton peak for resonant excitation is more than 5 times stronger compared to the above-resonant pump, further supporting the dominant role of exciton-exciton scattering process.

Supplementary Note 6: Pump-probe data

Evolution of the transient spectra at long delays

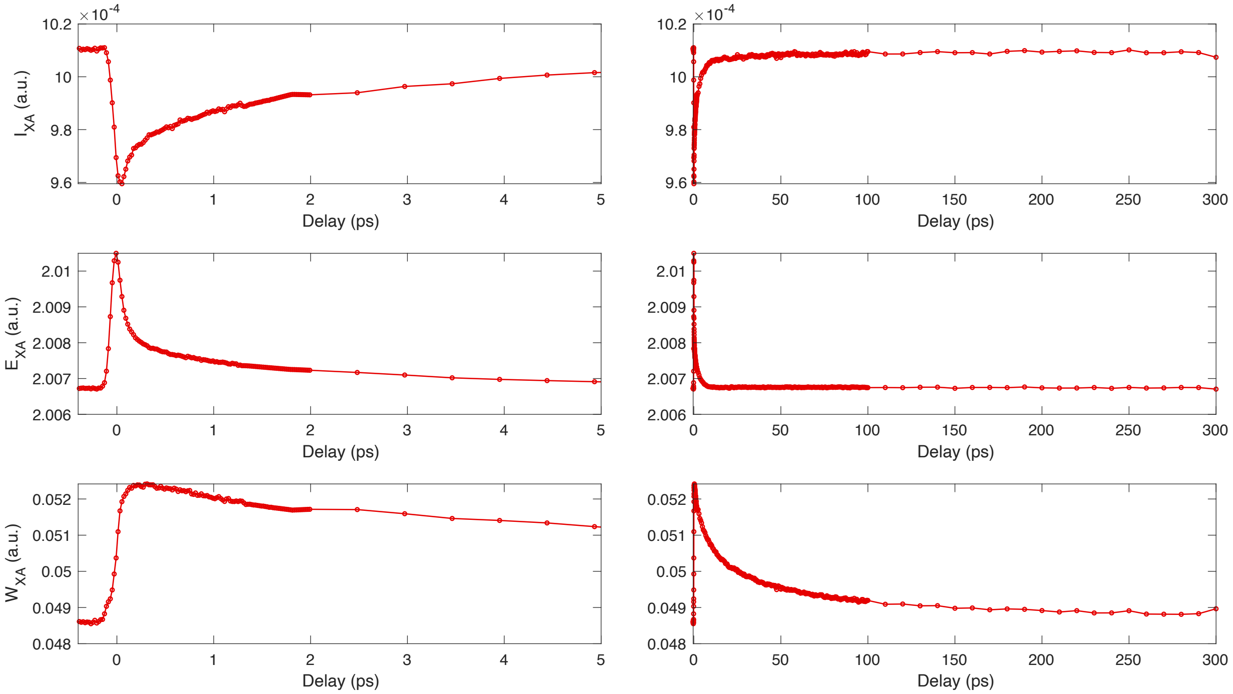

Figure S8: A exciton parameters. Evolution of the parameters of the A exciton lorentzian peak (intensity I_{XA}, energy E_{XA} and linewidth W_{XA}) as a function of the pump-probe time delay.

B exciton dynamics

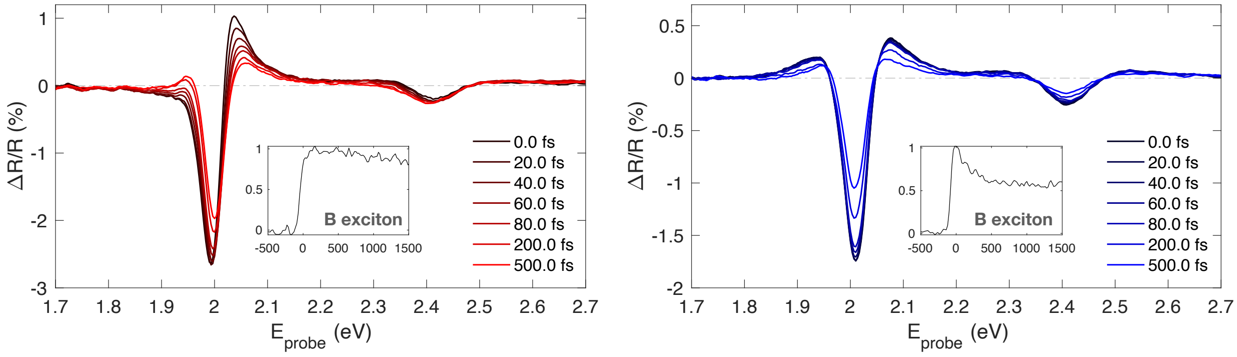

Figure S9: B exciton dynamics. Evolution of the transient spectra following excitation at the A(left) and B (right) exciton resonances. In the insets the dynamics of the B exciton.

Supplementary Note 7: Fitting the temporal dynamics

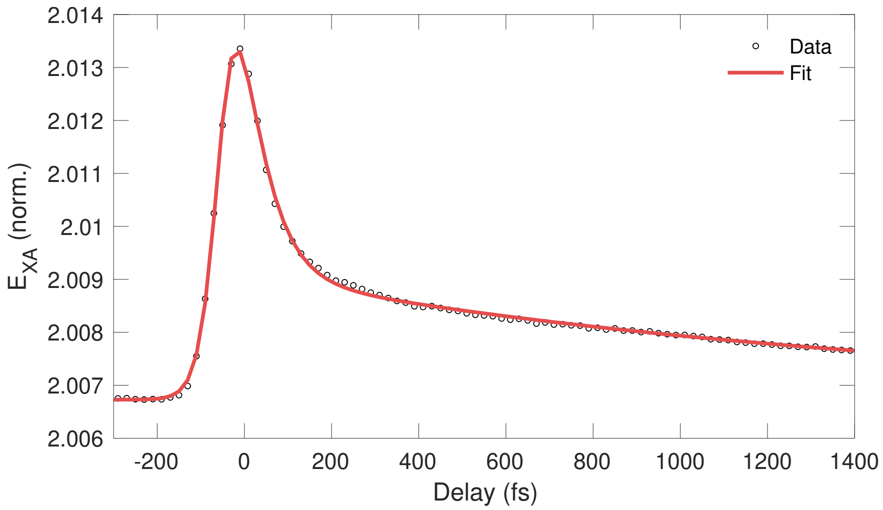

Figure S10: Temporal dynamics. Temporal evolution of the energy (E_{XA}) parameter of the A exciton lorentzian peak as a function of the pump-probe time delay. Here the pump is tuned on resonance to the A exciton (2.00 eV) and it has a fluence of $20\,\mu\mathrm{J/cm}^2$. The data (circles) are fitted to the sum of two exponential decay, convoluted with a Gaussian pulse (FWHM = 90 fs) which accounts for the temporal resolution of the experiment.

Supplementary Note 8: Theoretical calculations

All matrix elements are given in Ref.[17] Biexcitons and exciton-exciton scattering continua described in the main text by Eq. (2) are obtained by diagonalizing the two-exciton correlations on a basis of coupled 1s, 2s, and 3s excitons. The dynamics for exciton states higher than 3s excitons and continuum states is described by optically driven oscillations solely, which are characterized by the first two lines of Eq. (1) in the main text. The coupling of these higher states to the 1s to 3s states is numerically evaluated for Pauli blocking and Hartree–Fock interactions, corresponding to the second and third term on the right hand side of Eq. (1) in the main text.

The corresponding many-body contributions in Eq. (1) have been extensively studied in Ref.[17] Here we found that Coulomb mediated exciton-(bi)exciton interactions, *i.e.*, Hartree-Fock contributions (energy shifts) and exciton-biexciton correlations (excitation induced broadening due to biexcitonic continua) dominate the transient optical response in the coherent regime. In fact, Coulomb mediated exciton-exciton interactions are more than one order of magnitude more pronounced than the Pauli blocking contribution. Coulomb screening effects, due to the dielectric environment, are taken into account in the screened Coulomb potential term of the Wannier equation as extensively explained in Ref.[17] Spin and momentum dark states are not included in our calculations since they are barely occupied during ultrafast coherent excitation as their formation timescale is longer than 100 fs.[18,19]

Used parameters for monolayer WS_2 on a SiO_2 substrate: lattice constant $a_{2D} = 0.319\,\text{nm}$,[20] thickness $d_{2D} = 0.630$ nm,[20] single particle band gap $\varepsilon_g = 2.28$ eV adapted that the exciton energies match the experimentally measured values, effective electron mass $m^e_{K,\uparrow} = 0.27\ m_0$, $m^e_{K,\downarrow} = 0.36\ m_0$,[21] effective hole mass $m^h_{K,\uparrow} = 0.36\ m_0$, $m^h_{K,\downarrow} = 0.50\ m_0$,[21] in-plane dielectric constant $\epsilon_{2d} = 11.5$,[22] $\epsilon_{\text{SiO}_2} = 3.9$, plasmon peak energy $E_{2D} = 22.8$ eV,[22] effective hopping integral $t_{2D} = 1.37$ eV,[11] phonon-mediated linewidth broadening of A excitons $\Gamma_A = 27$ meV and B excitons $\Gamma_B = 62$ meV adapted to the experimentally measured linewidth. The intensity FWHM of the pump and probe pulse is 90 fs and 1 fs in the simulations, respectively.

References

(1) Liu F. et al. Disassembling 2D van der Waals crystals into macroscopic monolayers and reassembling into artificial lattices. *Science* **367**, 903–906 (2020)

(2) Y. Li & T. F. Heinz, Two-dimensional models for the optical response of thin films, *2D Mater.* **5**, 025021 (2018).

(3) Sie, E. J. *et al.* Observation of Exciton Redshift-Blueshift Crossover in Monolayer WS_2. *Nano Lett.* **2017**, *17*, 4210-4216.

(4) A. B. Kuzmenko, Kramers–Kronig constrained variational analysis of optical spectra. *Rev. Sci. Instrum.* **76**, 083108 (2005).

(5) Stancik, A. L.; Brauns, E. B. A simple asymmetric lineshape for fitting infrared absorption spectra. *Vibrational Spectroscopy* **47**, 66–69 (2008).

(6) F. Katsch, M. Selig & A. Knorr, Exciton-Scattering-Induced Dephasing in Two-Dimensional Semiconductors, *Phys. Rev. Lett.* **124**, 257402 (2020).

(7) M. Kira and S. W. Koch, Progress in quantum electronics **30**, 155 (2006).

(8) F. Katsch, M. Selig, A. Carmele, and A. Knorr, Physica Status Solidi (b) **255**, 1800185 (2018).

(9) M. Selig, G. Berghäuser, A. Raja, P. Nagler, C. Schüller, T. F. Heinz, T. Korn, A. Chernikov, E. Malić, and A. Knorr, Nature Communications **7**, 13279 (2016).

(10) F. Lengers, T. Kuhn, and D. E. Reiter, Physical Review B **101**, 155304 (2020).

(11) D. Xiao, G.-B. Liu, W. Feng, X. Xu, and W. Yao, Physical Review Letters **108**, 196802 (2012).

(12) A. Knorr, S. Hughes, T. Stroucken, and S. W. Koch, Chemical physics **210**, 27 (1996).

(13) F. Katsch and A. Knorr, Physical Review X **10**, 041039 (2020).

(14) W. Schäfer and M. Wegener, *Semiconductor optics and transport phenomena* (Springer Science & Business Media, 2013).

(15) R. Takayama, N. Kwong, I. Rumyantsev, M. Kuwata-Gonokami, and R. Binder, The European Physical Journal B-Condensed Matter and Complex Systems **25**, 445 (2002).

(16) S. Schumacher, G. Czycholl, and F. Jahnke, Physical Review B **73**, 035318 (2006).

(17) F. Katsch, M. Selig, and A. Knorr, 2D Materials **7**, 015021 (2020).

(18) Selig, M.; Berghäuser, G.; Richter, M.; Bratschitsch, R.; Knorr, A.; Malic, E. Dark and bright exciton formation, thermalization, and photoluminescence in monolayer transition metal dichalcogenides. *2D Materials*, *5*, 035017 (2018).

(19) M. Selig, F. Katsch, R. Schmidt, S. M. de Vasconcellos, R. Bratschitsch, E. Malić, and A. Knorr, Physical Review Research **1**, 022007(R) (2019).

(20) F. A. Rasmussen and K. S. Thygesen, The Journal of Physical Chemistry C **119**, 13169 (2015).

(21) A. Kormányos, G. Burkard, M. Gmitra, J. Fabian, V. Zólyomi, N. D. Drummond, and V. Fal'ko, 2D Materials **2**, 022001 (2015).

(22) A. Kumar and P. Ahluwalia, Physica B: Condensed Matter **407**, 4627 (2012).
